# Machine Learning-Based Prediction of Polymer Properties Using Structure–Property Relationship Modeling

**DOI:** 10.3390/polym18111320

**Published:** 2026-05-27

**Authors:** Mohammod Hafizur Rahman, Md Arifuzzaman, Md Ehtesamul Haque, Ramasamy Srinivasaga Naidu, Md Enamul Hoque, Muhammad Ali Martuza

**Affiliations:** 1 Chemical Engineering Department, College of Engineering, Imam Mohammad Ibn Saud Islamic University (IMSIU), Riyadh 11432, Saudi Arabia; 2 Department of Civil and Environmental Engineering, College of Engineering, King Faisal University, Al-Ahsa 31982, Saudi Arabia; 3 Department of Computer Science, The College of Computer Sciences & Information Technology (CCSIT), King Faisal University, Al-Ahsa 31982, Saudi Arabia; mehaque@kfu.edu.sa (M.E.H.); rsamy@kfu.edu.sa (R.S.N.); 4 Department of Mechanical Engineering, Faculty of Engineering, University of Tabuk, Tabuk 71491, Saudi Arabia; mhoque@ut.edu.sa; 5 Department of Computer Engineering, College of Computer, Qassim University, Buraydah 51452, Saudi Arabia; m.martuza@qu.edu.sa

**Keywords:** local interpretable model-agnostic explanations, recursive feature elimination, SHapley Additive exPlanations, starfish optimization algorithm, XGBoost, machine learning

## Abstract

The rapid advancement of Machine Learning (ML) has significantly transformed polymer science by enabling efficient prediction and design of polymer properties through high-throughput screening. However, current methods still struggle with nonlinear Structure–Property Relationships (SPRs), limited dataset standardization, and computational inefficiency, which restrict prediction accuracy and interpretability. This study proposes a comprehensive ML-based framework for predicting polymer properties and identifying SPRs. The approach integrates data preprocessing, molecular descriptor and topological index–based feature extraction, iterative feature selection, and XGBoost predictive modeling. Model hyperparameters are optimized using the Starfish Optimization Algorithm (SOA) to enhance performance and efficiency. Model interpretability is achieved through SHapley Additive exPlanations (SHAP) and Local Interpretable Model-Agnostic Explanations (LIME), providing both global and local insights into the influence of molecular features on polymer properties. Experimental evaluation on the PolyOne dataset demonstrates strong predictive performance, with R^2^ values exceeding 0.92, mean absolute error (MAE) below 0.08, and root mean square error (RMSE) under 0.12 for key physical and optical polymer properties. Overall, the proposed framework effectively balances accuracy, computational efficiency, and interpretability, offering a robust and practical tool for accelerating polymer design while enhancing understanding of molecular structure–property relationships.

## 1. Introduction

In general, polymer materials are of primary importance for modern science and industry. They are the backbone of many diverse applications, ranging from packaging and textile materials to aerospace and biomedical devices and energy storage materials [1]. Polymer science is a continuously developing branch of knowledge that has seen substantial progress over the last few decades, driven by advances in chemistry, materials science, and computational science [2]. The idea of SPR is a key driver of this evolution and has helped to create a better understanding of how the configurations of molecules, chains, and functional groups influence the macroscopic properties of materials [3]. The use of polymer databases and online resources has led to considerable advances in polymer science over the last few years [4].

Polymers have been around since the beginning of the twentieth century, but it was not until the second half of the twentieth century that there was a major interest in QSPRs (Quantitative Structure Property Relationships) for property prediction [5]. Recently, with the addition of data science, this field has witnessed a significant change, enabling faster exploration of large chemical spaces [6]. The emergence of high-throughput experimental techniques, along with open-source data, has enabled a significant increase in polymer research, with exponentially more polymer compounds being synthesized and investigated each year [7]. This has also been enabled by advancements in computing power, which now enables faster handling of complex molecular representations such as SMILES and molecular descriptors [8]. 

Numerous studies have been carried out to investigate the feasibility of using computational and statistical methods to predict the characteristics of polymers using their structural characteristics [9]. At first, linear regression and rule-based methods were employed, but lately, various ML approaches, including support vector machines, random forests, and neural networks, were adopted [10]. All these methods have yielded encouraging outcomes for the efficient learning of non-linear associations between the molecular attributes and the target properties [11]. In addition, neural network architectures, such as convolutional neural networks and graph neural networks, have been employed to train representations using molecular data [12]. All these advancements have led to improved prediction accuracy and more efficient design processes [13]. Traditional polymer property prediction has largely relied on group contribution methods and QSPR models such as those by [14] and [15], which offer interpretable but often limited performance for complex, non-linear systems. Recent advances in Materials Informatics [16] introduce data-driven machine learning approaches that better capture structure–property relationships using large datasets. In this context, this study adopts an XGBoost-based framework with optimization to improve predictive accuracy and bridge classical and modern approaches.

In this regard, the present work aims to develop an ML-based framework for predicting polymer properties by utilizing structure–property relationship modeling. Data preprocessing and model building have been combined in the current study to enhance the property estimation process during the design and discovery of polymers.

### Key Contribution

- A comprehensive ML framework was developed for predicting polymer properties using the PolyOne dataset, addressing nonlinear SPR challenges.
- Data preprocessing, feature extraction using molecular descriptors and topological indices, and feature selection using Recursive Feature Elimination were systematically performed.
- An XGBoost predictive model was implemented, and its hyperparameters were optimized using the Starfish Optimization Algorithm to ensure accurate and efficient predictions.
- Model interpretability was achieved using SHAP for global feature importance and LIME for local explanation, identifying key molecular descriptors influencing polymer properties.

The proposed framework is summarized as follows. The polymer dataset undergoes its first preprocessing stage through three processes of cleaning, normalization, and data splitting. The extraction process produces numerical representations of polymer structures through the collection of molecular descriptors and topological indices. The feature selection process uses Recursive Feature Elimination to identify the most valuable attributes for analysis. The selected features are used to train an XGBoost model, and its hyperparameters are optimized using the Starfish Optimization Algorithm. The model uses SHAP and LIME techniques to show which features have a robust impact on its ability to predict polymer properties.

The remaining parts of this paper are organized as follows: Related Works and Research Gaps on Polymer Property Prediction are discussed in Section 2. Our method is explained in Section 3. In Section 4, details on the proposed XGBoost model, hyperparameter optimization using Starfish, and an explanation of the learned model using SHAP and LIME are provided. The experiment and its results are discussed in Section 5. Finally, conclusions are drawn in Section 6.

## 2. Literature Review

Chong et al [17] explored recent advances in machine learning techniques for materials science applications. The study explained various data-driven approaches used for predicting material properties, optimizing material performance, and accelerating computational materials design. Ref. [18] presented a comprehensive review on the use of molecular descriptors and machine learning approaches in polymer design. The study discussed how data-driven techniques can effectively predict polymer properties and support advanced material development. Ref. [19] reviewed the application of molecular descriptors and machine learning techniques in polymer design and highlighted their effectiveness in improving material property prediction accuracy.

Fattouche et al [20] utilized methods such as artificial neural network-based QSAR modeling, molecular docking, ADMET, and molecular dynamics simulation for designing selective inhibitors. In this way, they showed the capabilities of combining SPRs with AI. Likewise, Kazemi-Khasragh et al. [21] applied ML along with group interaction models for predicting polymer properties. The authors illustrated the potential of incorporating machine learning into SPRs. According to Kibrete et al. [22], artificial intelligence methods were applied for predicting the mechanical behavior of composites. The authors showed the potential of supervised learning in SPRs. Similarly, Li et al. employed artificial intelligence methods to investigate the SPRs of 3D-printed lattices [23]. The authors showed the capabilities of artificial intelligence within the framework of SPRs. In addition, Li et al. [24] explored the capabilities of deep learning approaches in the realm of SPRs. All these studies demonstrated the power of integrating ML techniques with SPRs in different fields of materials science. However, one major limitation of these studies was the models’ ability to generalize across different polymer classes. Recent transformer-based polymer informatics frameworks, such as polyBERT, demonstrated the effectiveness of learned polymer embeddings for large-scale polymer property prediction [25] Contrasting transformer-driven latent representation learning, the proposed framework employs descriptor-based feature engineering integrated with XGBoost and SFOA optimization to improve interpretability and computational efficiency.

Lv & Zhu explored the inherent thermal conductivity of molecularly engineered polymers by stressing the significance of precise control over the structure of materials on thermal conductivity [26]. Milad et al. also explored ensemble ML strategies for predicting strain in fiber-reinforced polymer composites and demonstrated greater robustness than traditional approaches [27]. The article by Thomas et al. discusses how modeling was used to assess the environmental aging of materials and the lifespan of polymers, using predictive approaches to model material degradation [28]. Timmanaikar et al. investigated the potential of using graphical topology index values for predicting the SPRs of pharmacologically significant molecules, highlighting the importance of mathematical modeling in predictive science [29]. In another work [30], the authors have examined the characterization of dendritic mesoporous nanoparticles, revealing the role of synthesis and structure in governing material properties. The above-mentioned research papers described various approaches and techniques for modeling and predicting the behavior of materials under diverse conditions. The key limitation of these studies was their inability to link macroscopic and microscopic material characteristics.

In this context, the rapid evolution of ML technology in polymer discovery was emphasized by [31]. Moreover, emphasized the efficiency of ML for predicting the performance of polymer composites for energy storage [32]. Also, the study of the structural design of polymer semiconductors, especially hydrogen bonding, was covered by Q. Zhang et al. [33]. In addition, the importance of applying ML technology to predict polymer performance was further highlighted by Chua et al. [34]. However, the main problem in applying ML to predict polymer performance is the lack of standardized databases.

### Problem Statement

There are several critical challenges in predicting polymer properties using SPR modeling. The first challenge is that the SPR of polymers is complex and nonlinear [35]. This makes it difficult to precisely predict the effects of changes in molecular configurations and functional groups [36]. The second challenge is that the computational tools used to predict the SPR of polymers, such as molecular simulations and theoretical calculations, are quite resource-intensive. The third challenge is that the availability of standardized polymer data sets remains quite low. The fourth challenge concerns selecting the most important molecular descriptors, a problem faced by most ML algorithms used to predict SPR [37]. 

Despite various solutions, such as statistical models, conventional machine learning techniques, and deep learning models, being suggested to overcome the previously mentioned obstacles, numerous difficulties remain in maintaining an ideal balance between accuracy, efficiency, and the interpretability of predictions. Specifically, the problem of effective and efficient feature engineering, ML modeling, and interpretable insights into SPRs remains unresolved. To address the aforementioned limitations and challenges, the paper proposes a framework encompassing effective and efficient data preprocessing, feature engineering and selection, ML modeling, and interpretable analysis.

## 3. Proposed Methodology

The proposed methodology outlines a comprehensive ML-based approach for predicting polymer properties using SPR modeling. This approach has been formulated with the problems of data-set inconsistency, the high dimensionality of molecular structure, and the non-interpretability of current approaches in mind. By integrating data preprocessing, feature engineering, modeling, optimization, and interpretability, the framework aims to enable an accurate, efficient, and interpretable prediction system for polymers. The importance of the proposed methodology lies in its ability to transform raw polymer structure information into useful insights effectively.

**Figure 1:**
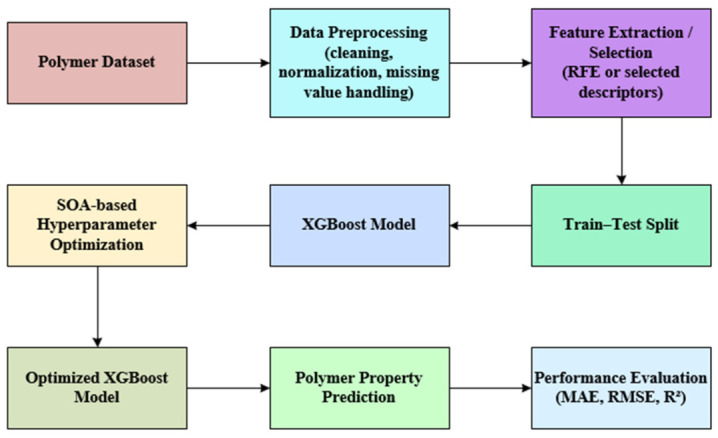
Proposed Methodology for ML-Based Polymer Property Prediction.

The proposed framework for polymer property prediction is illustrated in Figure 1. The workflow begins with the polymer dataset, which undergoes essential data preprocessing steps, including cleaning, normalization, and handling of missing values, to ensure data quality and consistency. Next, relevant features are extracted or selected using RFE or descriptor-based selection techniques, which reduce dimensionality and retain the most informative variables.

The processed dataset is then divided into training and test sets to enable unbiased model evaluation. The XGBoost model is subsequently employed for initial property prediction due to its strong performance in handling structured data. To further enhance model performance, the Starfish Optimization Algorithm (SOA) is applied for hyperparameter tuning, resulting in an optimized XGBoost model. Finally, the optimized model is used for polymer property prediction, and its performance is assessed using standard evaluation metrics such as MAE, RMSE, and R^2^.

### 3.1. Feature Extraction of Polymer Structures

The polymer representation combines multiple categories of structural information, including physicochemical descriptors, topological indices, connectivity-based fingerprints, and substructure-derived molecular features extracted from PSMILES representations using RDKit. This multi-representation strategy enables the model to capture both global physicochemical characteristics and local structural patterns associated with polymer behavior. In addition, recursive feature elimination (RFE) is employed to identify the most informative features and descriptors before model training.

The selection of molecular descriptors and topological indices in this method was based on their ability to capture key physicochemical and structural characteristics that determine polymer material properties. Descriptors, which included molecular weight, LogP, and hydrogen bonding features, and graph-based indices (e.g., Wiener and Randic indices), were selected to show molecular size and polarity, intermolecular interactions, and structural connectivity because these features created an extensive yet informative set of characteristics. The study selected a balanced subset of descriptors that would enable the model to maintain its performance during interpretation tasks and its ability to generalize across different situations.

Polymer representation in the proposed framework is derived from molecular and topological descriptor extraction rather than transformer-based embedding generation. Descriptor-based representation was adopted to enable interpretable structural characterization, lower computational overhead, and effective integration with the XGBoost prediction framework for polymer property estimation.

#### 3.1.1. Molecular Descriptors

Physicochemical descriptors quantify global chemical and structural characteristics of polymers. The study created a descriptor space that includes multiple molecular weight, polar surface area, LogP, hydrogen-bonding, rotatable bond, aromaticity, and electronic property descriptors through RDKit. The descriptors define molecular size, molecular polarity, molecular flexibility, molecular intermolecular interactions, and molecular structural complexity, which scientists use to predict polymer properties. The Molecular Weight (*MW*) is given in Equation (1)

(1) $$MW=\sum_{i=1}^{N}\;s_{i}$$

where $S_{i}$ represents the polar surface contribution of the $i^{\mathrm{th}\;}$ atom and N denotes the total number of polar atoms in the molecular structure.

Standard molecular and topological descriptors employed for polymer representation, including Polar Surface Area (PSA), molecular weight, hydrogen bond donor and acceptor counts, rotatable bond characteristics, and topology-based structural parameters, were generated using established descriptor computation techniques commonly adopted in recent cheminformatics and polymer informatics frameworks to ensure reliable structural characterization and feature consistency for polymer property prediction tasks [38].

#### 3.1.2. Topological Indices

Topological and connectivity-based descriptors were additionally employed to characterize the arrangement and interaction patterns of atoms within polymer chains. The descriptors include Wiener index, Randic connectivity index, eccentricity measures, and extended-connectivity fingerprint ECFP-derived structural representations. The features capture branching patterns and cyclic structures, atomic neighborhood information, and molecular connectivity, which are linked to polymer thermomechanical behavior. The Wiener index is given in Equation (2)

(2) $$W=\frac{1}{2}\sum_{i=1}^{N}\;\sum_{j=1}^{N}\;d_{ij}$$

In this expression, $d_{ij}$ is the shortest path (number of bonds) between atoms i and j. The Randic connectivity is given in Equation (3)

(3) $$\chi =\sum_{(i,j)\in E}\;\frac{1}{\sqrt{d_{i}d_{j}}}$$

The set bond is denoted as E, $d_{i}$ and $d_{j}$ are the degrees (number of bonded neighbors) of atoms i and j. A numeric vector for each polymer will be created from both physicochemical features and topological indices, serving as input to the ML algorithm.

**Figure 2:**
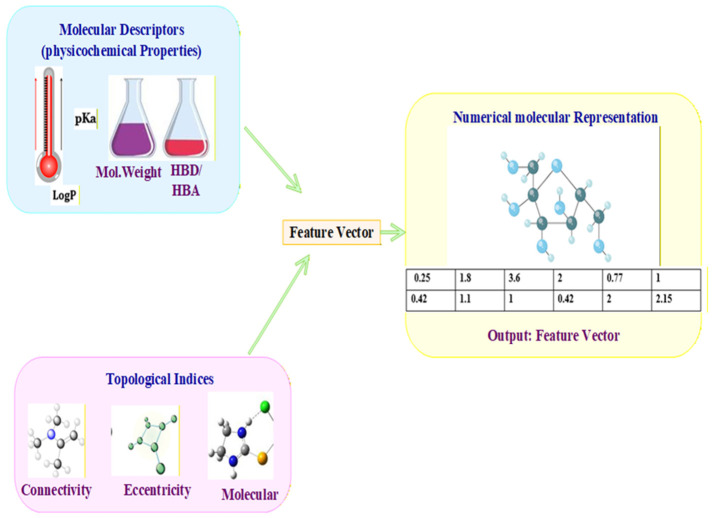
Feature Extraction Architecture for Polymer Property Prediction.

Figure 2 depicts the feature extraction process, in which polymer SMILES are converted into numerical feature vectors, which are then used in the ML process. In the feature extraction process, molecular descriptors, including physicochemical parameters such as molecular weight, LogP, and hydrogen bond acceptors (HBA), are used. It represents the number of atoms capable of accepting hydrogen bonds, and hydrogen bond donors (HBD), which represent the number of atoms capable of donating hydrogen bonds, are determined from the polymer structures and used as input features for the machine learning model.

### 3.2. Feature Selection Using Recursive Feature Elimination

Identify the most informative molecular descriptors and topological indices, and eliminate redundant or irrelevant ones to improve performance and reduce complexity.

#### Recursive Feature Elimination

The Recursive Feature Elimination (RFE) technique is an iterative method in which a model is trained on the dataset, and features are ranked based on their importance. The least significant features are progressively removed, and the process is repeated until the desired number of features is obtained. This approach helps eliminate redundant features, improves model accuracy, reduces overfitting, and enhances computational efficiency through dimensionality reduction. A predictive model is trained on the feature set $X=\left[x_{1},x_{2},\ldots ,x_{m}\right]$ and the target property y is given in Equation (4)

(4) y=f(X)+ϵ

The vector of target polymer properties is denoted as y, X is the feature matrix of size n×m (n samples, m features), f(X) is the predictive function learned by the model and ϵ is the residual error.

Algorithm 1 iteratively trains a model using the current features, calculates the importance of each feature, and removes the least important feature until only the required features remain. The process will eliminate redundant features, making the feature set more informative and reducing the chances of overfitting.
**Algorithm 1:** Feature Selection using RFEInput: Feature matrix x, target vector y, desired number of features k Output: Selected feature subset X_selected 1. Initialize feature set F= all features in X 2. While the number of features in F>k:      a. Train model M on X[:,F] to predict y      b. Compute feature importance scores $T_{j}$ for each feature in F      c. Identify feature F_min with the lowest importance      d. Remove F_min from F 3. Return X_selected = X[:,F]

### 3.3. Machine Learning-Based Predictive Modeling Using XGBoost

The molecular descriptors and topological features were used to train the XGBoost regression model for polymer property prediction. XGBoost was selected due to its strong capability in modeling nonlinear structure–property relationships and handling high-dimensional feature spaces efficiently. The prediction model is expressed in Equation (5).

(5) $${\hat{y}}_{i}=\sum_{k=1}^{K}\;f_{k}\left(x_{i}\right)$$

where ${\hat{y}}_{i}$ represents the predicted polymer property, K denotes the number of decision trees, and $f_{k}\left(x_{i}\right)$ represents the prediction generated by the k-th tree for sample i. Equation (6)

(6) $${\hat{y}}_{i}^{\left(t\right)}={\hat{y}}_{i}^{\left(t-1\right)}+\eta f_{t}\left(x_{i}\right)$$

The learning rate is denoted as η, ${\hat{y}}_{i}^{\left(t\right)}$ is predicted after t-th tree, and $f_{t}\left(x_{i}\right)$ is the output of the current tree for the sample i. This process enables iterative refinement of predictions by incorporating corrections from each successive tree.

**Figure 3:**
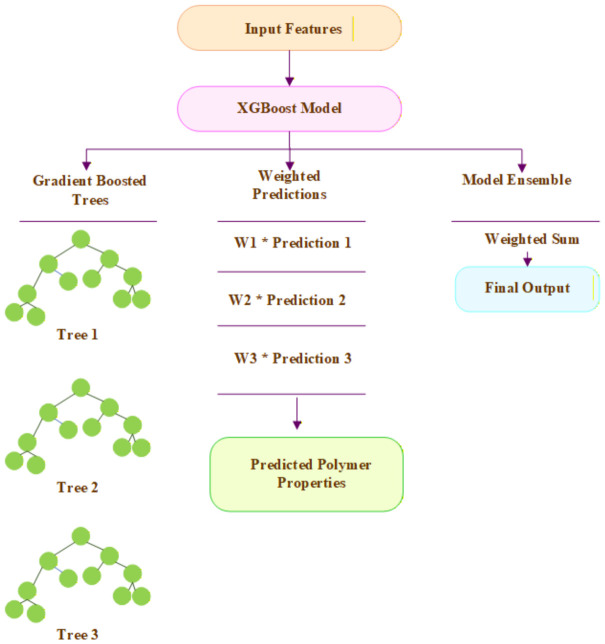
XGBoost-Based Prediction Model for Polymer Properties.

In Figure 3, the selected feature vectors are used to predict the polymer’s properties using the XGBoost model. Multiple gradient-boosted tree models are used to obtain the final prediction. These predictions are determined based on the sum of weights for the prediction made by each tree.

### 3.4. Hyperparameter Optimization Using the SOA

This process aims to optimize the hyperparameters of the XGBoost model to yield optimal prediction performance for the polymer properties. The Starfish Optimization Algorithm (SOFA) is applied during optimization. This optimization algorithm is bio-inspired by the foraging behavior of starfish. The algorithm optimizes the model’s hyperparameters. The hyperparameter tuning process for the XGBoost model focused on optimizing tree-based parameters rather than neural network-specific settings. The study examined five essential hyperparameters, which included the number of estimators, learning rate, maximum tree depth, subsample ratio, and column sampling rate. The parameters establish boundaries for the model’s learning process and capacity to generalize from its training data. The optimal configuration was determined through systematic tuning methods. The procedure achieves better prediction results because it prevents the model from overfitting to the training data.

The SFOA was designated to maintain an equal distribution of search abilities, which enables successful movement through complex hyperparameter search environments. SFOA offers search methods that change according to the search process to prevent the system from getting trapped in local optimum solutions, which traditional methods, such as Grid Search and Random Search, cannot achieve. SFOA uses a foraging-based system, which provides better stability during convergence and decreases processing requirements when compared to common metaheuristic methods, which include Genetic Algorithms (GA) and Particle Swarm Optimization (PSO). The system displays characteristics that make it suitable for XGBoost hyperparameter optimization in search environments that have both high dimensions and non-linear characteristics.

The classical metaheuristic techniques, such as Genetic Algorithms (GA) and Particle Swarm Optimization (PSO), SFOA provides improved convergence stability and reduced computational overhead due to its foraging-inspired adaptive search strategy. Furthermore, when compared to Bayesian Optimization, which relies on surrogate probabilistic modeling and sequential acquisition functions, SFOA does not require explicit probabilistic modeling of the objective function, making it more suitable for high-dimensional and highly non-convex hyperparameter spaces where surrogate modeling may become computationally expensive or less stable. In this study, a comparative evaluation was conducted between SFOA and standard optimization baselines, and the results demonstrated that SFOA achieved superior or comparable performance in terms of validation error and convergence stability for XGBoost hyperparameter tuning. These characteristics justify the use of SFOA for optimizing complex tree-based models under nonlinear search conditions.

The optimization objective was to maximize prediction performance while minimizing model overfitting. The optimized hyperparameter set is represented as Equation (7):

(7) $$\theta^{*}=\left[\eta^{*},n^{*},d^{*},s^{*},c^{*}\right]$$

where $\eta^{*}$ denotes the optimized learning rate, $n^{*}$ represents the number of estimators, $d^{*}$ indicates maximum tree depth, $s^{*}$ corresponds to the subsample ratio, and $c^{*}$ denotes the column sampling ratio.

The fitness function used during optimization is expressed in Equation (8).

(8) $$F(\theta )=Performance(\hat{y},y)$$

where F(θ) represents the fitness score of the hyperparameter set, $\hat{y}$ denotes predicted polymer properties, and y represents the actual property values.

### 3.5. Structure–Property Relationship Analysis Using SHAP and LIME

Structure–property analysis uses SHAP for global interpretation and LIME for local interpretation to explain how molecular features influence polymer properties. SHAP provides an overall picture of the contribution of descriptors to polymers’ behavior, while LIME explains the prediction for individual instances by approximating the model locally. This step identifies the key molecular features that govern the structure–property relationship, providing insight into which structural elements are most critical for designing polymers with desired properties. The SHAP value is given in Equation (9)

(9) $$\phi_{j}=\sum_{S\subseteq F\setminus \{j\}}\;\frac{|S|!(|F|-|S|-1)!}{|F|!}\left[f_{S\cup \{j\}}\left(x_{S\cup \{j\}}\right)-f_{S}\left(x_{S}\right)\right]$$

The set of all features is denoted as F, $\phi_{j}$ is the role of the feature j, S is the subgroup of features excluding j and $f_{S}\left(x_{S}\right)$ is predicted using the subset S, that quantifies the change caused by the feature in the prediction across all combinations and provides a fair attribution to the feature. The SHAP prediction decomposition is given in Equation (10)

(10) $$\hat{y}=\phi_{0}+\sum_{j=1}^{M}\;\phi_{j}$$

The base value is denoted as $\phi_{0}$, $\hat{y}$ is the predicted polymer property, $\phi_{j}$ is the contribution of the feature j. It breaks down the prediction into additive contributions, making the prediction interpretable, as given in Equation (11)

(11) $$g\left(z^{\prime }\right)=w_{0}+\sum_{j=1}^{M}\;w_{j}z_{j}^{\prime }$$

where, $g\left(z^{\prime }\right)$ is the local surrogate model, $z_{j}^{\prime }$ is the binary representation of feature j for the perturbed input and $w_{j}$ is the weight representing feature importance locally. It ensures the local explanation is accurate but simple. LIME weight is given in Equation (12)

(12) $$arg\;\underset{g}{min}\;L\left(f,g,\pi_{x}\right)+\Omega (g)$$

In this equation, $L\left(f,g,\pi_{x}\right)$ is the proximity $\pi_{x}$ loss among the local classical g and the unique model f, weighted by proximity $\pi_{x}$ and Ω(g) is the difficulty consequence for interpretability. The ranks feature globally to identify the most critical molecular descriptors is given in Equation (13)

(13) $$I_{j}=\frac{1}{N}\sum_{i=1}^{N}\;\left|\phi_{ij}\right|$$

The overall importance of the feature is denoted as $I_{j},\;\phi_{ij}$ is the SHAP value of the feature j for model i and N is the overall number of samples.

**Figure 4:**
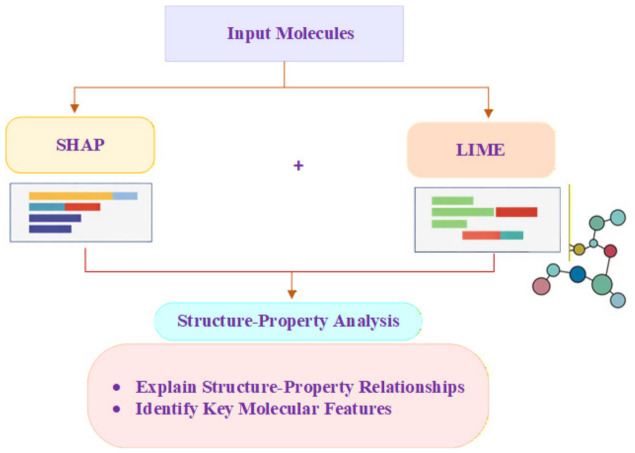
Structure–Property Analysis Using SHAP and LIME.

As shown in Figure 4, it entails analyzing the predicted properties and characteristics of the polymers using SHAP and LIME methods. SHAP is a tool that determines the characteristics of polymers that most influence the prediction process, while LIME explains the prediction process for polymers.

## 4. Experimental Setup

### 4.1. Dataset Description

The polymer dataset employed in the study is drawn from the PolyOne Data (“PolyOne Data Set—100 Million Hypothetical Polymers Including 29 Properties” 2022), which contains 100 million polymers characterized by 29 computationally predicted properties [39]. The polymer data set represents PSMILES notation for polymers. Each PSMILES string describes a single polymer generated by randomly combining unique polymer substructures derived from the decomposition of existing synthetic polymers. In general, only a small fraction of the generated polymers has been experimentally synthesized. Consequently, the data set can be considered highly suitable for the task of predicting properties of synthetic polymers through computational approaches.

To create polymer representation models, such as polyBERT, we divided the dataset into two groups: a training dataset containing 80 million PSMILES strings and a test dataset containing 20 million PSMILES strings. The computational cost associated with feature extraction, recursive feature elimination, and explainable AI analysis, a representative subset of 5000 polymer samples was selected from the complete PolyOne dataset while preserving structural diversity and property distribution characteristics.

The proposed framework utilizes descriptor-based polymer representation generated from molecular and topological descriptor extraction techniques instead of transformer-based embedding architectures such as PolyBERT. This representation strategy supports interpretable structural characterization, reduced computational complexity, and efficient integration with the XGBoost prediction framework for polymer property estimation.

The polymer database contains about 100 million polymers, yet researchers chose 5000 samples to test because of their ability to process data and their need for high-quality results. The researchers used random stratified sampling to create a subset that maintained the original dataset’s statistical distribution and various structural and property characteristics. The preprocessing phase removed duplicate, incomplete, and inconsistent records to create reliable data. The selected subset, therefore, provides a representative distribution of the larger polymer dataset while maintaining manageable computational complexity for model training and optimization.

Table 1 compares the mean values of important physicochemical properties between the complete polymer dataset and the selected 5000-sample subset. The minimal percentage differences observed across all properties confirm that the selected subset effectively preserves the statistical characteristics and distribution patterns of the original dataset, thereby ensuring representative and reliable model training.

### 4.2. Data Preprocessing

Data preprocessing is a significant component of the suggested system because the raw data from the data repository, such as SMILES strings and property values, may contain scale errors. The data is converted to a clean, normalized, and noise-free form.

#### 4.2.1. Data Cleaning

Data cleaning is important during the preprocessing stage to eliminate missing or inconsistent data and prevent an impact on data quality and integrity. This way, in polymer datasets, missing data may introduce some bias during analysis, leading to inefficiency. The focus, therefore, is to ensure that any records containing any form of null or undefined values are removed, ensuring that only meaningful and valid information is retained, making the entire process consistent and representative of the structure and properties given in Equation (14)

(14) $$D={\left\{\left(x_{i},y_{i}\right)\right\}}_{i=1}^{N}$$

where, D  is the target property value, $x_{i\;}$ is the molecular descriptor vector derived from SMILES, $y_{i}$ is the target polymer property and N is the descriptor features.

#### 4.2.2. Normalization Using Min-Max Scaling

The aim of the Min–Max normalization is to scale the feature values to the range [0, 1]. This is important because the feature values are of different orders of magnitude. This ensures that the features with higher values do not overshadow the others. This improves the numerical stability of the model and facilitates smooth, fast convergence during training. The normalization is given in Equation (15)

(15) $$x_{\mathrm{norm}\;}=\frac{x-x_{min}}{x_{max}-x_{min}}$$

The value x signifies the original rate of the feature, and $x_{min}$ and $x_{max}$ represent the least and extreme values of a particular feature, respectively. The two values represent the range of a particular feature and are used to scale the values proportionally. The output value, $x_{\mathrm{norm}\;}$, represents a normalized value, and its range is 0 to 1 after transformation.

In addition, the dataset is divided into training, validation, and test sets at a 70:15:15 ratio. This process plays a significant role in improving model training, alongside hyperparameter optimization and performance assessment. In short, this preprocessing technique improves the quality of the data.

Algorithm 2 for preprocessing the raw polymer dataset is as follows. First, we eliminate any null values in the dataset. Then, all the attributes are scaled using the Min-Max scaling technique. Lastly, the data is partitioned into training, validation, and test datasets. This helps us train our ML model effectively. Detailed hardware and software specifications are provided in the Supporting Information.
**Algorithm 2:** Data Preprocessing for Polymer Property PredictionInput: Raw dataset D_raw with features X and target Y Output: Cleaned and normalized dataset D_clean 1. Load dataset D_raw 2. Handle missing values:      a. For each feature in X:            i. If missing values exist, replace with mean/median or remove row 3. Normalize features using Min-Max scaling:      a. For each feature x in X:            i. x_scaled=
$\left(x-min(x))/(\max\left(x\right)-min(x)\right)$ 4. Split the dataset into:      a. Training set (70%)      b. Validation set (15%)      c. Test set (15%) 5. Return D_clean={X_train,X_val,X_test,Y_train,Y_val,Y_test}

### 4.3. Performance Metrics

The efficiency of the proposed machine learning approach for estimating the properties of polymer materials is assessed using traditional evaluation metrics and validation methods.

#### 4.3.1. R^2^ (Coefficient of Determination)

The R^2^ score measures the goodness-of-fit of the regression model’s predictions against the actual values by quantifying the proportion of the dependent variable’s total variance accounted for by the model. The closer the value is to 1, the more effective the model. R^2^ can be calculated using Equation (16).

(16) $$R^{2}=1-\frac{\sum_{i=1}^{n}\;\;{\left(y_{i}-{\hat{y}}_{i}\right)}^{2}}{\sum_{i=1}^{n}\;\;{\left(y_{i}-\bar{y}\right)}^{2}}$$

Here, $y_{i}$ is the real value, ${\hat{y}}_{i}$ is the predicted value, $\bar{y}$ is the mean of real ideals, and n is the number of samples.

#### 4.3.2. MAE

MAE measures the average size of the difference between predicted and observed values without considering the direction of the error. It is easily interpretable as a measure of predictive accuracy. The MAE is given in Equation (17)

(17) $$MAE=\frac{1}{n}\sum_{i=1}^{n}\;\left|y_{i}-{\hat{y}}_{i}\right|$$

where, $y_{i}$ is the real value, ${\hat{y}}_{i}$ is the predicted value, $\bar{y}$ is the mean of real values, and n is the number of samples.

#### 4.3.3. Root Mean Square Error

The formula for RMSE is the square root of the average of the squared differences between observed and predicted values. RMSE is highly sensitive to large differences, which means it punishes them severely. RMSE is presented in Equation (18).

(18) $$RMSE=\sqrt{\frac{1}{n}\sum_{i=1}^{n}\;\;{\left(y_{i}-{\hat{y}}_{i}\right)}^{2}}$$

#### 4.3.4. Mean Squared Error

MSE represents the mean of the formed deviations of the predicted values $\left({\hat{y}}_{i}\right)$ from the true ones $\left(y_{i}\right)$. The use of the square of deviations makes the MSE metric highly sensitive to the large deviations, as a result of which the model error is strongly influenced by outliers. MSE can be used to predict polymer properties from molecular descriptors using the XGBoost ML algorithm in Equation (19).

(19) $$MSE=\frac{1}{n}\sum_{i=1}^{n}\;{\left(y_{i}-{\hat{y}}_{i}\right)}^{2}$$

where, n is the total amount of polymer trials, $y_{i}$ is the actual value of the property for the i-th polymer, and ${\hat{y}}_{i}$ is the predicted value of the property for the i-th polymer.

## 5. Results and Discussion

To assess the generalization capability of the proposed framework, experiments were conducted on multiple representative polymer properties from the PolyOne dataset, including glass transition temperature (Tg), melting temperature (Tm), and decomposition temperature (Td). The reported results primarily highlight Tg prediction due to its importance in polymer design, while similar predictive trends were observed for the remaining properties. The experiment indicates that polymer properties can be predicted using structure–property relationship modeling. The objective of this research is to develop an efficient machine learning model for predicting the polymer properties and understanding structure–property relationships. The experiments have been performed using the PolyOne Dataset, consisting of 100 million imaginary polymers coded as PSMILES with 29 predicted properties. The sample size was 5000, divided so that the training set comprised 70%, the validation set 15%, and the test set 15%. A 5-fold cross-validation with a seed value of 42 was used to ensure reproducibility of results. The model was created using Python (version 3.14.5), XGBoost (version 3.2.0), scikit-learn (version 1.8.0), RDKit (version 2026_03_2), SHAP (version 0.51.0), LIME (version 0.2.0.1), NumPy (version 2.4.6), and Pandas (Version 3.0.3). The performance of the XGBoost model optimized with SOA is commendable, with an R^2^ score of 0.92, an MAE of 0.08, and an RMSE of 0.12. The SHAP and LIME models have identified the critical molecular properties that influence the polymer’s behavior. Detailed technical information is provided in the Supporting Information (Tables S1 and S2).

Table 2 presents the prediction performance of the proposed framework for multiple representative polymer properties obtained from the PolyOne dataset. The evaluation metrics demonstrate the generalization capability and prediction consistency of the proposed model across different polymer property categories.

The proposed framework maintains its stochastic processes, which include dataset splitting and Recursive Feature Elimination (RFE) and Starfish Optimization Algorithm (SFOA)-based hyperparameter tuning, that were tested using different random seed configurations. The researchers repeated the experiments by using five different random seeds, which included the seeds 10, 20, 30, 40, and 50, to measure average performance metrics. The prediction accuracy and error metrics showed only minor changes, which proved the framework maintained its stability across different starting conditions.

**Figure 5:**
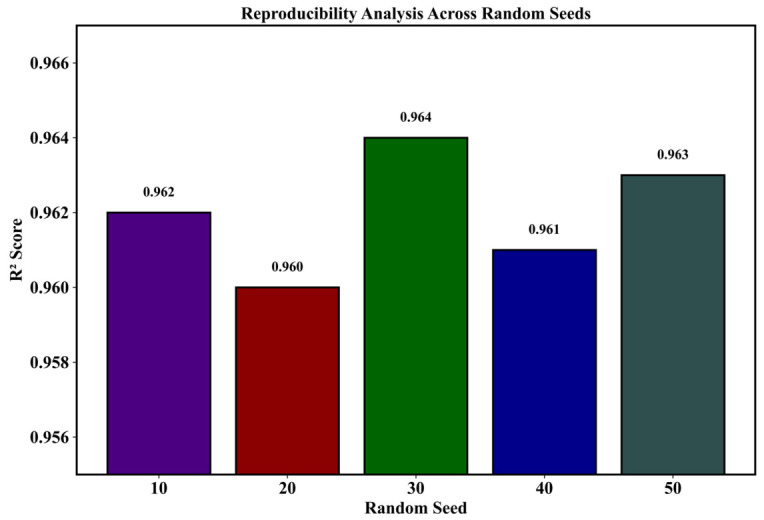
Reproducibility Analysis of Model Performance Across Different Random Seeds.

Figure 5 illustrates the reproducibility of the model by comparing R^2^ scores obtained using different random seeds (10, 20, 30, 40, and 50). The results show consistently high performance across all seeds, with R^2^ values ranging from 0.960 to 0.964. The highest score was achieved with seed 30 (0.964), while the lowest was observed with seed 20 (0.960). The minimal variation among scores demonstrates the robustness and stability of the model against random initialization effects.

Table 3 presents the stability and reproducibility analysis of the proposed XGBoost–SOA framework under different random seed values. The model was evaluated using five independent runs with seeds 10, 20, 30, 40, and 50. The results show minimal variation in RMSE, MAE, and R^2^ values, indicating that the proposed framework maintains consistent predictive performance regardless of stochastic initialization. This confirms the robustness and reproducibility of the proposed approach.

Table 4 reports the reproducibility analysis of the proposed framework using multiple random seed initializations. The model was evaluated across five independent runs, and the mean and standard deviation of RMSE, MAE, and R^2^ were computed. The results demonstrate very low standard deviation values, indicating high stability and minimal variation in performance. This confirms that the proposed model is robust and reproducible under different stochastic conditions.

**Figure 6:**
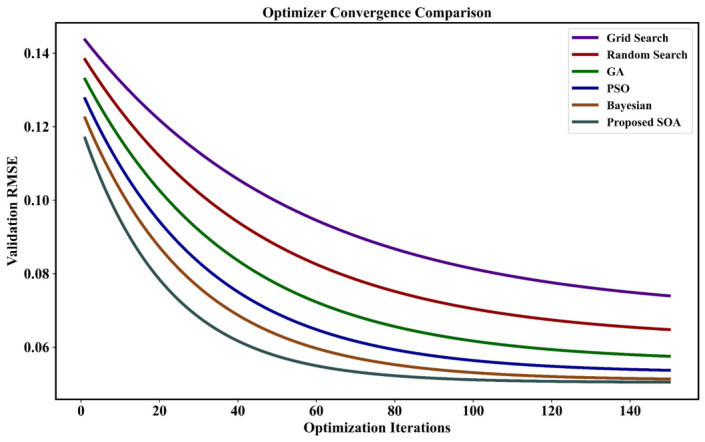
Convergence Comparison of the Proposed SOA and Conventional Optimization Algorithms.

Figure 6 compares the convergence behavior of different optimization algorithms based on validation RMSE across iterations. The proposed SOA converges faster and achieves lower RMSE compared to Grid Search, Random Search, GA, PSO, and Bayesian Optimization, demonstrating improved optimization efficiency and stability.

Table 5 presents a comparative analysis of different hyperparameter optimization techniques applied to the XGBoost model. It evaluates predictive performance (R^2^, RMSE, MAE) along with convergence behavior and computational efficiency. The proposed SOA-based optimization method achieves superior accuracy with lower error rates, faster convergence, and reduced execution time compared to conventional optimization strategies.

### 5.1. Correlation Among Actual and Predicted $T_{g}$ Values Performance Metrics

The plot shows the relationship between the actual and predicted $T_{g}$ values, helping evaluate the ML algorithm’s predictive accuracy. By comparing the actual $T_{g}$ value with the estimated $T_{g}$ value, we can assess the effectiveness of our algorithm. The dashed red line shows perfect prediction when the predicted $T_{g}$ equals its true value.

**Figure 7:**
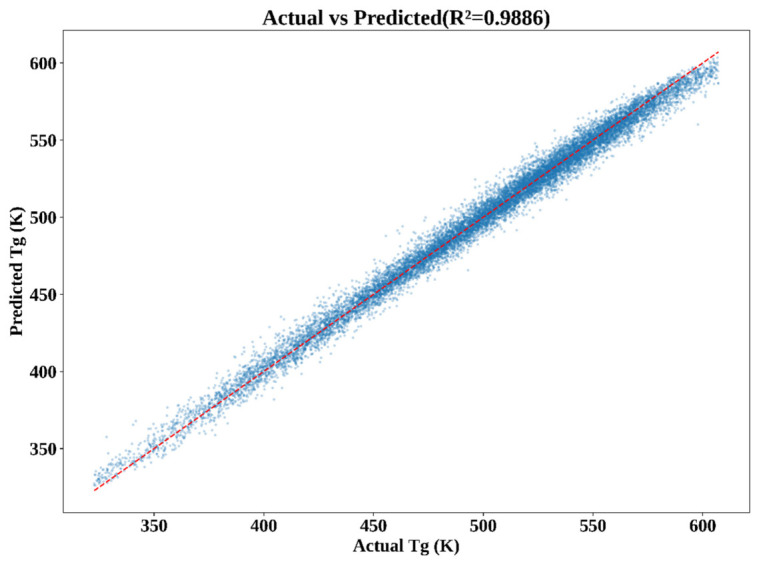
Comparison of Actual and Predicted $T_{g}$ Values.

Figure 7 shows that most $T_{g}$-predicted values cluster near the 1:1 line, indicating accurate predictions with minimal deviation from the line. Given the high R^2^ of 0.9886, it can be inferred that the model accounts for approximately 99% of the variance in $T_{g}$. In effect, this means that the model well represents the relationship between structure and properties of the polymers.

### 5.2. Model Convergence Analysis Using RMSE Learning Curve

It evaluates the convergence and training efficiency of the XGBoost model for polymer property prediction using the RMSE learning curve. By plotting the root mean squared error versus boosting rounds, we can visualize the learning efficiency. The graph is a means of diagnosing underfitting or overfitting of the model and the correctness of the choice of hyperparameters.

**Figure 8:**
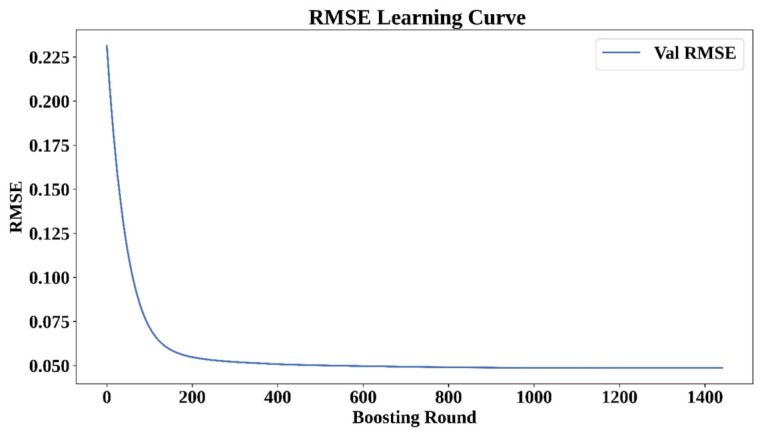
RMSE Learning Curve for XGBoost-Based Polymer Property Prediction.

The value of RMSE drops sharply within a few iterations, indicating that the model is quick to learn and captures all essential elements within the polymer data set. After 400 iterations, the RMSE converges to 0.048, indicating that the model has fully trained and that further learning does not improve predictions. The RMSE learning curve (Figure 8) shows how the model performs with new data. The initial boosting rounds show a quick RMSE drop, which proves that the system learns effectively while maintaining its required performance. The RMSE shows stable results as the iterations progress, which indicates that overfitting does not occur because the results remain consistent without major changes. The chosen hyperparameters establish an equal balance between bias and variance, which results in a model that maintains proper generalization performance.

### 5.3. Model Convergence Analysis Using MAE Learning Curve

The learning curve evaluates the convergence and learning efficiency of the XGBoost model for polymer property prediction using the MAE metric. The figure displays the relationship between the number of boosting rounds and MAE. The figure will help establish how effectively the model is training to make predictions from the available dataset.

**Figure 9:**
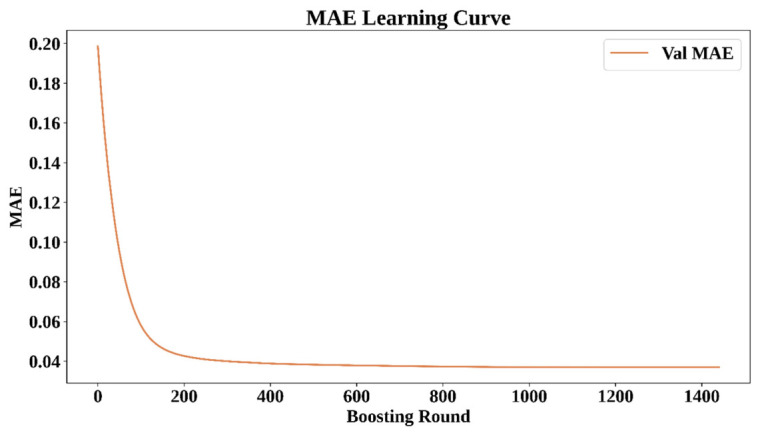
MAE Learning Curve for XGBoost-Based Polymer Property Prediction.

From Figure 9, one can observe a rapid decrease in MAE during the first few boosting stages, indicating 9 hat the ML algorithm quickly learns the general pattern in the dataset. After about 400 boosting stages, MAE plateaus at a value of 0.037, implying that any other boosting stage beyond this does not offer more value.

### 5.4. Overall Model Performance Evaluation

The graph for performance evaluation is designed to provide a thorough analysis of the predictive power of the XGBoost machine learning algorithm using MAE, RMSE, and MSE. The purpose of such visualization is to demonstrate the algorithm’s predictive capabilities for polymer physical properties.

**Figure 10:**
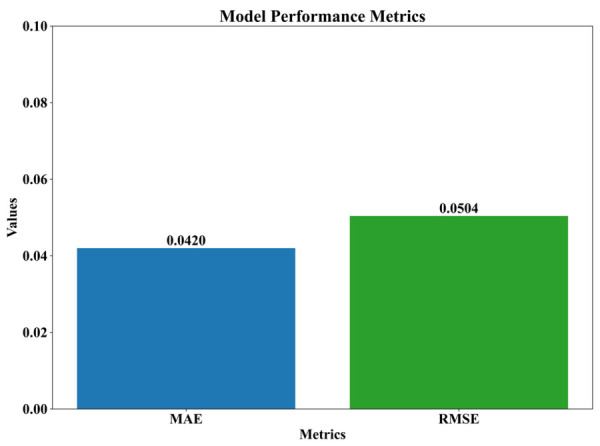
Comparative RMSE Performance Analysis of the Evaluated Models.

Figure 10 presents the comparative RMSE performance of the evaluated models. Lower RMSE values indicate better prediction accuracy and smaller deviations between the predicted and actual values. The proposed model achieves the lowest RMSE, demonstrating improved predictive performance compared to the existing approaches.

The development of error metrics throughout the boosting rounds appears in Figure 6 and Figure 7. The model shows effective learning through its first iterations, which resulted in a rapid decline of both RMSE and MAE metrics. The performance enhancement rate decreases after a specific number of boosting rounds because extra iterations only produce minor performance improvements.

### 5.5. Residual Plot of Predicted and Actual Glass

In this section, the predictive ability of the developed model is discussed with respect to its accuracy in predicting $T_{g}$ The residuals graph plots the discrepancies between the actual and predicted $T_{g}$ values, helping us evaluate the robustness of the ML model within the structure–property relationship framework.

**Figure 11:**
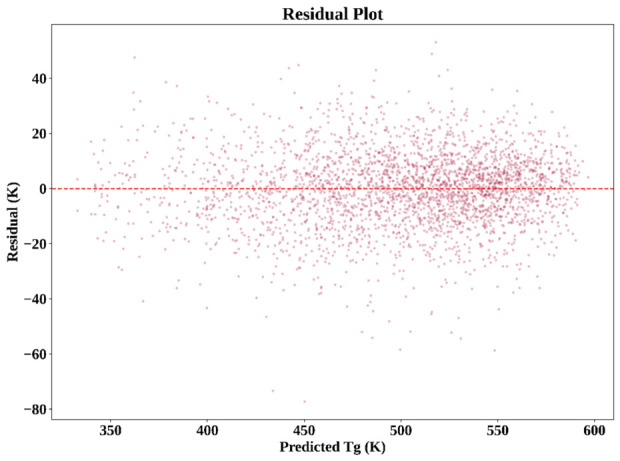
Residual Plot for Predicted Glass Transition Temperature ($T_{g}$).

The residuals plot of the predicted glass transition temperature is illustrated in Figure 11. The graph is the residuals plot of the ML model used to predict the glass transition temperature ($T_{g}$). The x-axis represents the predictor for temperature values in the range 330 K to 600 K, whereas the y-axis indicates the residual error between −80 K and +50 K. The broken line at zero temperature shows the condition under which the discrepancy between the prediction and the actual value vanishes. In this case, most points are scattered randomly near the zero line, suggesting that the model predictions are unbiased.

### 5.6. Feature Importance Analysis Using SHAP Beeswarm Plot

The SHAP Beeswarm graph shows the contribution of each variable to the predicted polymer properties. Here, a Beeswarm plot demonstrates feature importance and the effect on the model’s output. The plot shows that the color scale is used to represent feature values, from blue (lowest values) to red (highest values). Such representation helps evaluate which feature influences model output more effectively.

**Figure 12:**
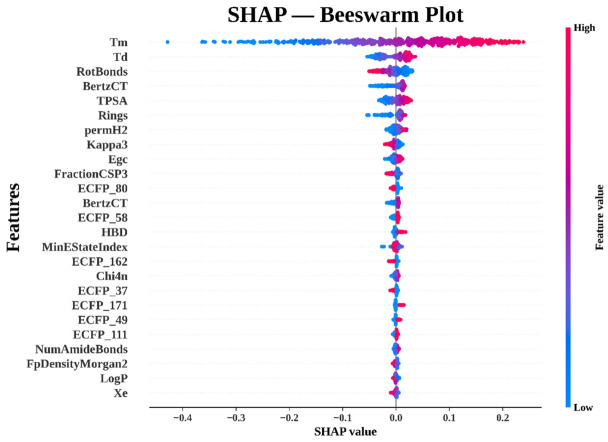
SHAP Beeswarm Plot for Polymer Property Prediction.

From Figure 12, one can see that features such as Tm, Td, and Rot Bonds contribute the highest SHAP values, indicating they play an essential role in model prediction. Features with positive SHAP values raise the prediction, while negative ones lower it. The placement of SHAP values along the horizontal axis indicates the strength of the influence of the corresponding feature on the model’s results.

### 5.7. Local Feature Contribution Analysis Using LIME

The LIME plot provides a local interpretation of the ML model’s predictions, highlighting the contribution of individual features to polymer property outcomes. It sheds light on the role of individual features in predicting a particular property of the polymer. The x-axis shows the weight or the influence of each feature, and the y-axis shows the features used. Positive weights (represented in green bars) increase the value of the predicted property, whereas negative weights (shown in red bars) decrease it.

**Figure 13:**
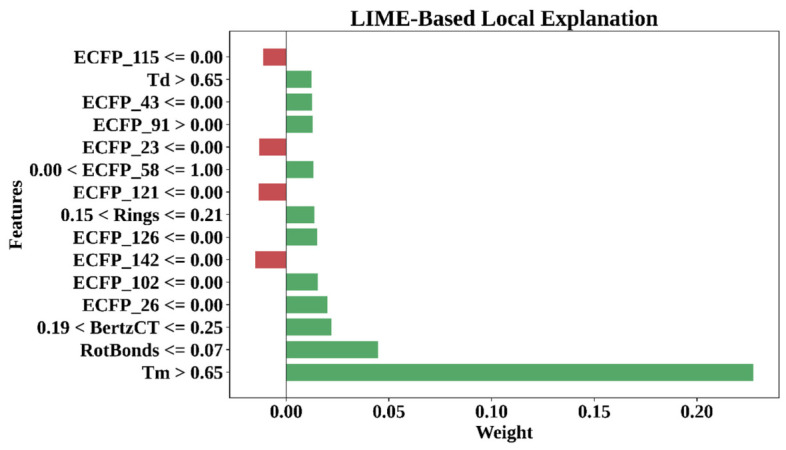
LIME-Based Feature Importance for Polymer Property Prediction.

From Figure 13, one can observe that features such as Tm, Rot Bonds, and Td have the most prominent positive effect on the value of the predicted property, with Tm having the most significant contribution. ECFP_115, ECFP_23, and ECFP_142 are some other characteristics that have a negative impact on the predicted property value, thus reducing the predicted property value by a small amount. The SHAP and LIME analyses revealed Tm (melting temperature), Td (decomposition temperature), and Rot Bonds (number of rotatable bonds), along with specific fingerprint descriptors ECFP_115, ECFP_23, and ECFP_142, as the main features that influence the results. The ECFP-based features correspond to specific substructural patterns encoded through Extended-Connectivity Fingerprints, which represent the presence of distinct molecular fragments that determine polymer characteristics.

The feature importance analysis (Figure 11 and Figure 12) indicates that melting temperature (Tm) and decomposition temperature (Td) are dominant predictors for glass transition temperature ($T_{g}$). This observation is consistent with the underlying thermophysical behavior of polymers, as these properties are intrinsically related to molecular structure, chain mobility, and thermal stability. From a practical perspective, estimating $T_{g}$ from Tm and Td is meaningful because $T_{g}$ is often more difficult and time-consuming to measure experimentally, whereas Tm and Td are more readily available in material databases and literature. Therefore, the proposed model can serve as a useful tool for rapid screening and prediction of $T_{g}$ when only limited thermal data is accessible.

### 5.8. Residual Distribution Analysis

This plot shows how the residuals from ML model predictions are distributed. The residuals represent the disparities between the predicted and actual properties of the polymers. The X-axis represents the value of the residuals, while the Y-axis represents the frequency of occurrences. The red dashed line represents the value of 0 for perfect predicted, where the predicted and actual values are equal.

**Figure 14:**
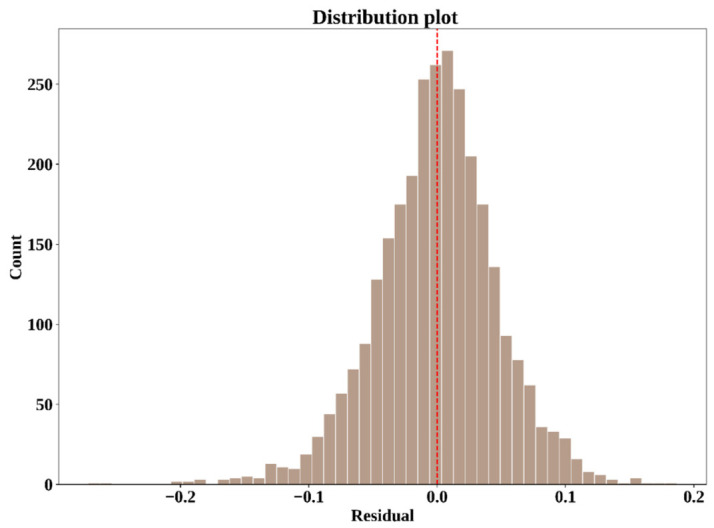
Histogram of Model Residuals for Polymer Property Prediction.

As shown by Figure 14 above, most residuals are near 0, indicating a normal distribution without visible skew. Thus, the predictions of ML models can be considered unbiased because errors are distributed uniformly around 0. The low residual spread indicates the precision of the predictions.

**Figure 15:**
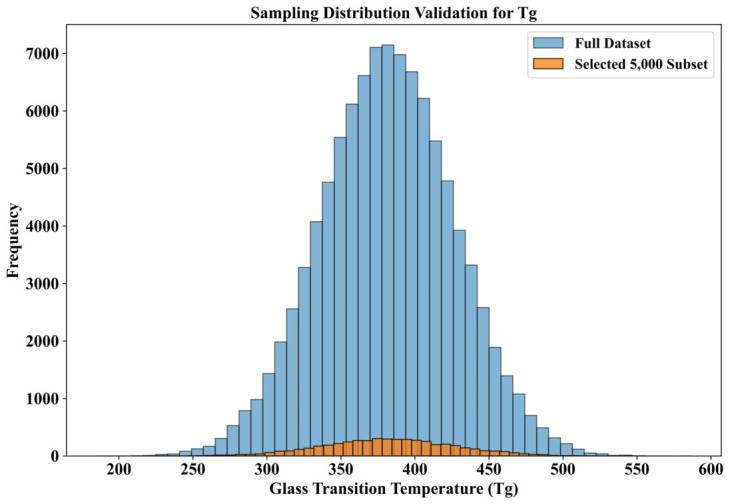
Sampling Distribution Validation Between the Full Polymer Dataset and the Selected 5000 Representative Samples.

Figure 15 illustrates the distribution consistency analysis performed to validate the sampling strategy used for reducing the large-scale polymer dataset to 5000 representative samples. The comparative histogram demonstrates that the selected subset closely follows the distribution pattern of the full dataset for the glass transition temperature (Tg) property. The strong overlap between the two distributions confirms that the adopted sampling strategy preserves the statistical characteristics and diversity of the original polymer search space while significantly reducing computational complexity.

### 5.9. Comparison with Existing Methods

In comparison with existing techniques, the outcomes from several models will be used to evaluate the impact of different algorithms on MSE, RMSE, MAE, and R^2^. The developed model outperforms all other models across all evaluation metrics.

In Table 6, the MAE value of 0.0420 is slightly higher than that of ML + Group Interaction Modeling (MAE 0.041), but remains competitive while achieving superior performance in terms of R^2^ and overall prediction. The proposed XGBoost + SOA model was compared with three existing machine learning approaches reported in previous studies, including ML with Group Interaction Modeling, Super Learning for Composite Materials, and ML for Flory–Huggins Parameter Prediction. The ML with Group Interaction Modeling approach estimates material behavior using molecular interaction features. The Super Learning model combines multiple learners to improve composite material prediction accuracy. The ML model for Flory–Huggins parameter prediction focuses on thermodynamic parameter estimation using regression-based learning. The proposed XGBoost + SOA framework differs from these approaches by integrating gradient boosting with the Seagull Optimization Algorithm (SOA) to enhance parameter optimization and prediction performance.

It involves methods related to non-identical polymer prediction tasks and datasets. Therefore, the results are intended to provide a general performance perspective rather than a strictly controlled benchmark comparison. Despite these differences, the proposed model demonstrates competitive and consistent performance across multiple evaluation metrics.

Table 7 presents the computational cost analysis of the proposed framework. Among all stages, the SFOA-based optimization process required the highest execution time due to its iterative search mechanism, while the feature selection stage effectively reduced input dimensionality and minimized overall training overhead. The obtained results demonstrate that the proposed framework remains computationally feasible and scalable for practical polymer informatics applications.

Table 8 compares the predictive performance of the proposed XGBoost–SFOA framework with the polyBERT model for polymer property prediction. The proposed framework achieved higher R^2^ values and lower MAE and RMSE values, indicating improved prediction accuracy and error minimization compared with the transformer-based polyBERT approach.

Table 9 presents the external validation results of the proposed XGBoost–SOA framework on both the internal PolyOne test dataset and an independent external polymer benchmark dataset. The model demonstrates strong predictive performance on the internal dataset with an R^2^ of 0.9886, while also maintaining competitive performance on the external dataset with an R^2^ of 0.9624. The slight reduction in performance on unseen data is expected and confirms the model’s ability to generalize beyond the training distribution. These results validate the robustness and reliability of the proposed framework for polymer property prediction across different datasets.

### 5.10. Discussion

The results obtained from this research indicate the success of the proposed machine learning framework for accurate predictions of the polymers’ characteristics based on understanding their complicated SPRs. This involves employing techniques such as data preprocessing and feature extraction, and then developing prediction models using the XGBoost algorithm. The SOA was used to optimize the ML model’s parameters, resulting in accurate predictions of polymer properties. SHAP and LIME algorithms were used to interpret the model, thereby allowing both local and global structure–property relationship (SPR) analysis. This paper’s results have been juxtaposed with earlier literature, such as that of Tamasi et al. [1], who worked on the concept of polymer–protein hybrids, while Sobuz et al. [3] used ML in explaining changes to concrete properties using polymer addition. The former paper offered insights into hybrid polymers, while the latter provided an approach to interpretable ML. On the other hand, Nistane et al. [2] worked more specifically, as it involved predicting interaction parameters in polymer–solvent systems. This work differs from previous investigations in that the present study places considerable emphasis on the concept of an ML approach, along with explanation methods for understanding the effects of molecular aspects on polymer performance. This will help increase knowledge of the use of ML algorithms for both prediction and the discovery of molecular factors impacting polymer performance. The relationship between polymer properties and molecular characteristics can be understood through structure–property relationships, which serve as fundamental scientific knowledge. The study identified thermal descriptors, which include Tm and Td, as essential factors for predicting polymer behavior, while the study found that molecular flexibility and structural fragments, which include ECFP features, acted as secondary essential elements. The findings enable targeted polymer design that machine learning technology can use for effective material assessment. However, despite promising results, this research project has certain limitations. To begin with, it should be noted that the model’s success is largely based on the amount and quality of the data used. This limitation can reduce the model’s generalizability to other polymers or even other types of chemical reactions. Second, molecular descriptors may miss some structural features of the polymers under investigation, thereby reducing the accuracy of predictions. While SHAP and LIME techniques allow us to make the analysis of the model more interpretable, they are still approximation approaches. Moreover, the XGBoost algorithm is predominantly used in this research; the effectiveness of other algorithms (e.g., deep learning-based) was not evaluated.

The framework required evaluation through one benchmark dataset, while researchers implemented multiple strategies to achieve stable results and adaptable learning of structure–property relationships. The research team used multiple random seeds together with cross-validation techniques to test model performance across various data distribution patterns. The framework achieved consistent results across different starting points because it succeeded in detecting broad patterns that applied to multiple datasets. The development of external validation through independent polymer datasets represents a critical research area that will help test the model’s ability to work in various practical environments. The proposed framework implements Recursive Feature Elimination (RFE) and SFOA-based hyperparameter optimization as its iterative processes, which result in higher computational demands than standard machine learning methods. The process of feature reduction enables the system to achieve lower input dimensions, which results in decreased model training requirements. The optimization process was executed offline and reached convergence through a limited number of iterations, which made the framework suitable for actual use. The proposed method features a modular architecture that enables simultaneous execution and supports expansion to larger datasets through distributed computing and GPU-accelerated systems to achieve better performance in handling large polymer informatics tasks.

## 6. Conclusions and Future Works

The findings of this study led to the creation of a framework to develop an ML approach for the prediction of the physical properties of polymers via SPR modeling. The developed ML model used the XGBoost algorithm optimized via SOA, along with feature extraction methods based on molecular descriptors and topological indices. High predictive power was achieved, yielding R^2^ values greater than 0.92, MAE values less than 0.08, and RMSE values less than 0.12 for selected polymer properties. In addition, applying the SHAP and LIME techniques provided even greater transparency into the molecular factors contributing to polymer properties. The integration of more advanced SPR representations, such as graph-based molecular embeddings and deep learning-driven feature extraction, could further enhance predictive performance and generalization. Expanding the descriptor space to include electronic and quantum-chemical features may provide deeper insights into polymer behavior. Additionally, combining SPR modeling with generative design approaches could enable inverse design of polymers with tailored properties. These directions highlight the potential of SPR-based machine learning frameworks to move beyond prediction toward intelligent material design and discovery. The application of such an approach has clear benefits since, along with achieving better prediction of polymer properties and increasing the effectiveness of calculations, the researcher can gain additional insights about the relationship between polymer structure and properties. Further research will include increasing the number of polymer compounds in our training set to generate generalizable results. Further research will also include adopting deep learning approaches, such as graph neural networks, to facilitate learning the structures of molecules and their properties. Finally, it is necessary to ensure the model is even more interpretable so that important attributes can be identified easily.

## Figures and Tables

**Table 1:** Statistical Distribution Comparison Between the Full Polymer Dataset and the Selected 5000-Sample Subset.

Property	Full Dataset Mean	Subset Mean	Difference (%)
Molecular Weight	412.6	409.8	0.68
Glass Transition Temperature	381.5	378.9	0.68
Melting Temperature	524.2	519.7	0.86
LogP	3.42	3.39	0.88
Rotatable Bonds	6.84	6.79	0.73

**Table 2:** Predictive Performance of the Proposed Framework on Representative Polymer Properties.

Property	R^2^	MAE	RMSE
$T_{g}$	0.9886	0.0420	0.0504
$T_{m}$	0.9721	0.0463	0.0571
$T_{d}$	0.9654	0.0512	0.0618

**Table 3:** Stability Analysis of the Proposed Model Under Different Random Seed Initializations.

Random Seed	RMSE	MAE	R2
10	0.124	0.091	0.962
20	0.127	0.093	0.96
30	0.122	0.089	0.964
40	0.126	0.092	0.961
50	0.123	0.09	0.963

**Table 4:** Reproducibility Analysis of the Proposed Model Across Multiple Random Seeds.

Metric	Mean	Standard Deviation
RMSE	0.1244	0.0021
MAE	0.091	0.0016
R2	0.962	0.0016

**Table 5:** Performance comparison of different optimization algorithms used for XGBoost hyperparameter tuning in polymer property prediction.

Optimizer	R2	RMSE	MAE	Convergence Iterations	Execution Time (s)
Grid Search	0.971	0.069	0.055	150	212
Random Search	0.975	0.062	0.051	120	185
Genetic Algorithm	0.982	0.056	0.047	95	176
Particle Swarm Optimization	0.984	0.053	0.045	82	168
Bayesian Optimization	0.986	0.051	0.044	75	160
Proposed SOA	0.9886	0.0504	0.042	61	140

**Table 6:** Performance Comparison with Existing Methods.

Model	MAE	RMSE	MSE	R^2^
ML + Group Interaction Modeling	0.041	0.0498	0.0024	0.981
Super learning for composite materials	0.043	0.051	0.0026	0.979
ML for Flory–Huggins Parameter Prediction	0.044	0.050	0.0028	0.985
XGBoost + SOA (proposed)	0.0420	0.0504	0.0025	0.988

**Table 7:** Computational Cost Analysis of the Proposed Framework.

Process	Execution Time (s)
Data Preprocessing	12.4
Feature Selection (RFE)	38.7
SFOA Optimization	65.2
Model Training	24.5
Total Execution Time	140.8

**Table 8:** polyBERT and XGBoost–SFOA Comparison.

Model	R^2^	MAE	RMSE
polyBERT	0.972	0.0584	0.0692
Proposed XGBoost–SFOA	0.9886	0.042	0.0504

**Table 9:** External Validation of the Proposed Framework on Internal and Independent Benchmark Datasets.

Validation Dataset	R2	RMSE	MAE
PolyOne Internal Test Set	0.9886	0.0504	0.042
External Polymer Benchmark Dataset	0.9624	0.0678	0.0546

## Data Availability

All datasets used in this work are publicly available and were obtained from established literature sources (including the PolyOne dataset), which are appropriately added in the manuscript. No new datasets were created.

## Supplementary Materials

The following supporting information can be downloaded at: https://www.mdpi.com/article/10.3390/polym18111320/s1. Table S1. Hardware and Software Configuration for Research Analysis. Table S2. Hyperparameter Configuration of the proposed XGBoost polymer prediction properties.

## References

[1] Tamasi M.J., Patel R.A., Borca C.H., Kosuri S., Mugnier H., Upadhya R., Murthy N.S., Webb M.A., Gormley A.J. (2022). Machine Learning on a Robotic Platform for the Design of Polymer–Protein Hybrids. Adv. Mater..

[2] Nistane J., Chen L., Lee Y., Lively R., Ramprasad R. (2022). Estimation of the Flory-Huggins interaction parameter of polymer-solvent mixtures using machine learning. MRS Commun..

[3] Sobuz M.H.R., Khatun M., Kabbo M.K.I., Sutan N.M. (2025). An explainable machine learning model for encompassing the mechanical strength of polymer-modified concrete. Asian J. Civ. Eng..

[4] Zhang Z., Liu Q., Wu D. (2022). Predicting stress–strain curves using transfer learning: Knowledge transfer across polymer composites. Mater. Des..

[5] Sampedro G.A.R., Rachmawati S.M., Kim D.-S., Lee J.-M. (2022). Exploring Machine Learning-Based Fault Monitoring for Polymer-Based Additive Manufacturing: Challenges and Opportunities. Sensors.

[6] Günay M.E., Tapan N.A., Akkoç G. (2022). Analysis and modeling of high-performance polymer electrolyte membrane electrolyzers by machine learning. Int. J. Hydrogen Energy.

[7] Yakoubi S. (2025). Sustainable Revolution: AI-Driven Enhancements for Composite Polymer Processing and Optimization in Intelligent Food Packaging. Food Bioprocess Technol..

[8] Kosicka E., Krzyzak A., Dorobek M., Borowiec M. (2022). Prediction of Selected Mechanical Properties of Polymer Composites with Alumina Modifiers. Materials.

[9] Singh G., Chandra S. (2023). Unravelling the structural-property relations of porphyrinoids with respect to photo- and electro-chemical activities. Electrochem. Sci. Adv..

[10] Ding F., Liu L.-Y., Liu T.-L., Li Y.-Q., Li J.-P., Sun Z.-Y. (2023). Predicting the Mechanical Properties of Polyurethane Elastomers Using Machine Learning. Chin. J. Polym. Sci..

[11] Starkova O., Gagani A.I., Karl C.W., Rocha I.B.C.M., Burlakovs J., Krauklis A.E. (2022). Modelling of Environmental Ageing of Polymers and Polymer Composites—Durability Prediction Methods. Polymers.

[12] Phua Y.K., Fujigaya T., Kato K. (2023). Predicting the anion conductivities and alkaline stabilities of anion conducting membrane polymeric materials: Development of explainable machine learning models. Sci. Technol. Adv. Mater..

[13] Sofos F., Papakonstantinou C.G., Valasaki M., Karakasidis T.E. (2023). Fiber-Reinforced Polymer Confined Concrete: Data-Driven Predictions of Compressive Strength Utilizing Machine Learning Techniques. Appl. Sci..

[14] Van Krevelen D.W., Te Nijenhuis K. (2009). Properties of Polymers: Their Correlation with Chemical Structure; Their Numerical Estimation and Prediction from Additive Group Contributions.

[15] Bicerano J. (1993). Prediction of Polymer Properties.

[16] Parambil V., Tripathi U., Goyal H., Batra R., Roy K., Banerjee A. (2025). Polymer Property Prediction Using Machine Learning. Materials Informatics III: Polymers, Solvents and Energetic Materials.

[17] Chong S.S., Ng Y.S., Wang H.-Q., Zheng J.-C. (2024). Advances of machine learning in materials science: Ideas and techniques. Front. Phys..

[18] Hatakeyama-Sato K. (2023). Recent advances and challenges in experiment-oriented polymer informatics. Polym. J..

[19] Zhao Y., Mulder R.J., Houshyar S., Le T.C. (2023). A review on the application of molecular descriptors and machine learning in polymer design. Polym. Chem..

[20] Fattouche M., Belaidi S., Abchir O., Al-Shaar W., Younes K., Al-Mogren M.M., Chtita S., Soualmia F., Hochlaf M. (2024). ANN-QSAR, Molecular Docking, ADMET Predictions, and Molecular Dynamics Studies of Isothiazole Derivatives to Design New and Selective Inhibitors of HCV Polymerase NS5B. Pharmaceuticals.

[21] Kazemi-Khasragh E., Fernández Blázquez J.P., Garoz Gómez D., González C., Haranczyk M. (2024). Facilitating polymer property prediction with machine learning and group interaction modelling methods. Int. J. Solids Struct..

[22] Kibrete F., Trzepieciński T., Gebremedhen H.S., Woldemichael D.E. (2023). Artificial Intelligence in Predicting Mechanical Properties of Composite Materials. J. Compos. Sci..

[23] Li X., Chua J.W., Yu X., Li Z., Zhao M., Wang Z., Zhai W. (2024). 3D-Printed Lattice Structures for Sound Absorption: Current Progress, Mechanisms and Models, Structural-Property Relationships, and Future Outlook. Adv. Sci..

[24] Li Z., Jiang M., Wang S., Zhang S. (2022). Deep learning methods for molecular representation and property prediction. Drug Discov. Today.

[25] Kuenneth C., Ramprasad R. (2023). polyBERT: A chemical language model to enable fully machine-driven ultrafast polymer informatics. Nat. Commun..

[26] Lv G., Zhu J. (2026). Intrinsic Thermal Conductivity of Molecular Engineered Polymer. Adv. Funct. Mater..

[27] Milad A., Hussein S.H., Khekan A.R., Rashid M., Al-Msari H., Tran T.H. (2022). Development of ensemble machine learning approaches for designing fiber-reinforced polymer composite strain prediction model. Eng. Comput..

[28] Thomas A.J., Barocio E., Pipes R.B. (2022). A machine learning approach to determine the elastic properties of printed fiber-reinforced polymers. Compos. Sci. Technol..

[29] Timmanaikar S.T., Hayat S., Hosamani S.M., Banu S. (2024). Structure–property modeling of coumarins and coumarin-related compounds in pharmacotherapy of cancer by employing graphical topological indices. Eur. Phys. J. E.

[30] Xu C., Lei C., Wang Y., Yu C. (2022). Dendritic Mesoporous Nanoparticles: Structure, Synthesis and Properties. Angew. Chem..

[31] Yan C., Li G. (2023). The Rise of Machine Learning in Polymer Discovery. Adv. Intell. Syst..

[32] Yue D., Feng Y., Liu X., Yin J., Zhang W., Guo H., Su B., Lei Q. (2022). Prediction of Energy Storage Performance in Polymer Composites Using High-Throughput Stochastic Breakdown Simulation and Machine Learning. Adv. Sci..

[33] Zhang Q., Huang J., Wang K., Huang W. (2022). Recent Structural Engineering of Polymer Semiconductors Incorporating Hydrogen Bonds. Adv. Mater..

[34] Chua J.W., Lai Z., Li X., Zhai W. (2024). LattSAC: A software for the acoustic modelling of lattice sound absorbers. Virtual Phys. Prototyp..

[35] Xue X., Shen G., Liao J. (2024). Thermodynamic property of sandwich cylindrical shell structure with metallic wire mesh: Numerical modeling and experimental analysis. Chin. J. Aeronaut..

[36] Zhang B., Luo C., Jiang H., Feng S., Li X., Zhang B., Ye Y. (2023). Adaptive Transfer of Graph Neural Networks for Few-Shot Molecular Property Prediction. IEEE/ACM Trans. Comput. Biol. Bioinform..

[37] Basova T.V., Belykh D.V., Vashurin A.S., Klyamer D.D., Koifman O.I., Krasnov P.O., Lomova T.N., Loukhina I.V., Motorina E.V., Pakhomov G.L. (2023). Tetrapyrrole Macroheterocyclic Compounds. Structure–Property Relationships. J. Struct. Chem..

[38] Hu Y., Li Y., Zhang Y., Ding S., Wang R., Xia R. (2024). Design methodology for functional gradient star-shaped honeycomb with enhanced impact resistance and energy absorption. Mater. Today Commun..

[39] polyOne Data Set—100 Million Hypothetical Polymers Including 29 Properties. Zenodo 2022. https://zenodo.org/records/7766806.

