# Supplementary material for: Machine Learning-Based Prediction of Polymer Properties Using Structure–Property Relationship Modeling
Source: Polymers (Basel). 2026 May 27;18(11):1320. doi: 10.3390/polym18111320 (PMC13259003; doi:10.3390/polym18111320)
Supplement: Supplementary file 1 [file polymers-18-01320-s001.zip › polymers-4298945-supplementary/polymers-4298945-supplementary.pdf]

Supporting information

Hardware and software settings for our investigation are listed in Table S1 below.

Table S1. Hardware and Software Configuration for Research Analysis.

Category	Component	Specification/Version
**Software**	scikit-learn	1.4.0
	SOA	custom
	RDKit	2023.9
	SHAP	0.44.0
	LIME	0.2.0.1
	Pandas	2.1.4
	NumPy	1.26.3
	Pyarrow	14.0.1
	Scipy	1.11.4
**Hardware**	CPU Model	Intel Core i7-12700H
	CPU Max Boost	4.70 GHz
	RAM Capacity	16 GB DDR5
	GPU Model	NVIDIA GeForce RTX 3060 (Laptop)
	Storage Type	NVMe SSD
	Storage Capacity	512 GB
	Storage Read Speed	3500 MB/s

The hyperparameter Table S2 used for the optimization of T_g prediction using the XGBoost algorithm was fine-tuned for optimal accuracy and stability of the model. These comprise the total number of boosting rounds used at 2450, maximum depth at 8, learning rate at 0.0312, and row and column subsampling at 0.82 and 0.75-0.72, respectively. Regularisation is implemented using Min_child_weight is 3, and Max_delta_step is 2. Base_score = 0.5 is used as an initial prediction value. Feature selection and tuning were done using RFE for 150 features, 50 iterations, and 5-fold cross-validation. A Min-Max scaler was employed for input normalization.

Table S2. Hyperparameter Configuration of the proposed XGBoost polymer prediction properties.

Model	Hyperparameter	Value
**N_estimators**	Amount of Boosting Trees	2450
**Max_depth**	Extreme Tree Depth	8
**Learning_rate**	Learning Rate η	0.0312
**Subsample**	Row Subsampling Ratio	0.82

**Colsample_bytree**	Column Fraction per Tree	0.75
**Colsample_bylevel**	Column Fraction per Level	0.68
**Colsample_bynode**	Column Fraction per Node	0.72
**Min_child_weight**	Minimum Sum of Hessian in Leaf	3
**Max_delta_step**	Max Delta Step per Tree	2
**Base_score**	Initial Prediction Score	0.5
**N_jobs**	Parallel CPU Threads	-1
**N_features**	Features Selected via RFE	150
**N_trials**	Optimisation Trials	50
**Cv_folds**	Cross-Validation Folds	5
**Scaler**	Feature & Target Scaler	Min Max Scaler [0,1]
**Rfecv_step**	RFE Step Size	0.05
**Rfecv_cv**	RFE Cross-Validation Folds	3
**Rfecv_scoring**	RFE Scoring Metric	R2
